# Supplementary figures and images for: Self-suppressing behavioral patterns and depressive traits exacerbate chronic pain: Psychological trait assessment using the structured association technique method
Source: PLoS One. 2025 Mar 17;20(3):e0319647. doi: 10.1371/journal.pone.0319647 (PMC11913282; doi:10.1371/journal.pone.0319647)

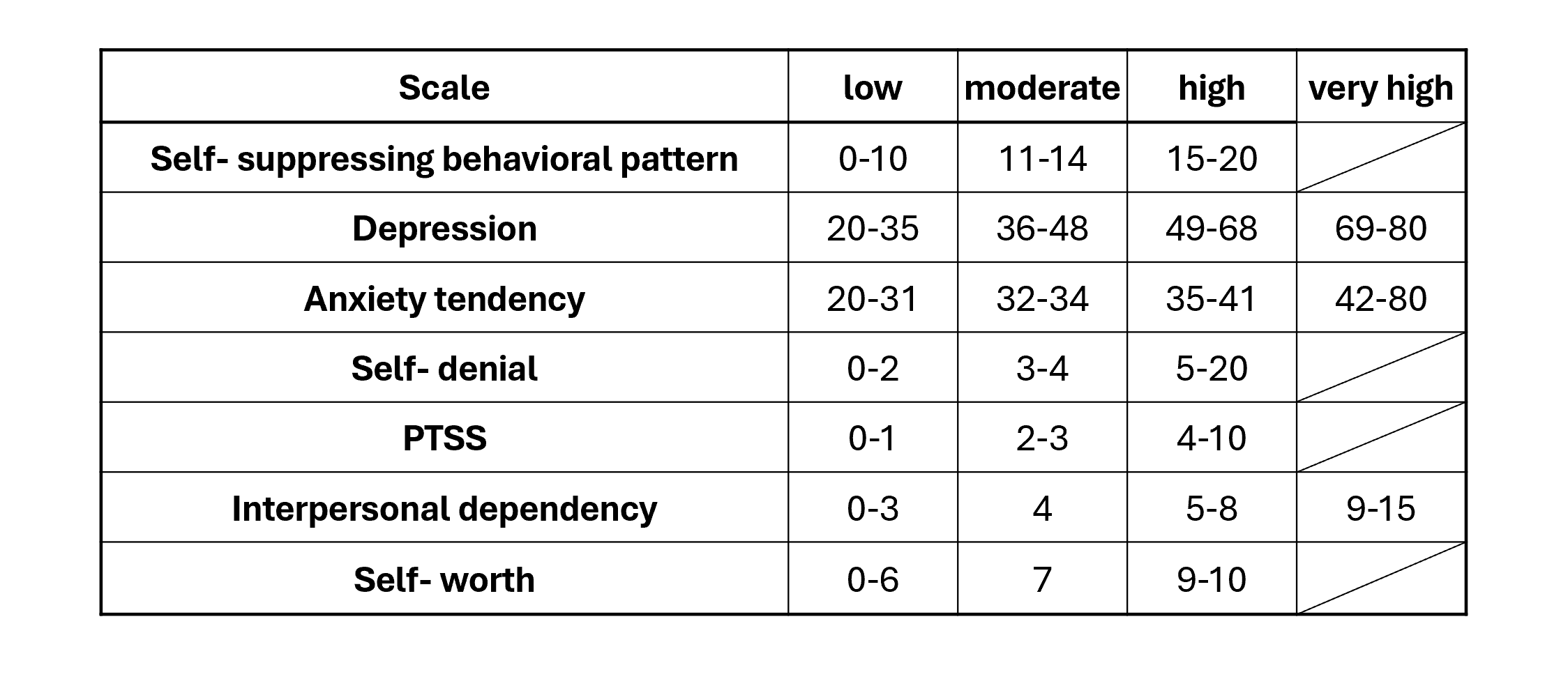

Supplement: S1 Table — The score ranges in the low, moderate, and high levels of each scale (the self-suppressing behavioral pattern scale, the depression scale, the anxiety tendency scale, the self-denial scale, the PTSS scale, the interpersonal dependency scale, and the self-worth scale) are shown. (TIF) [file pone.0319647.s001.tif]

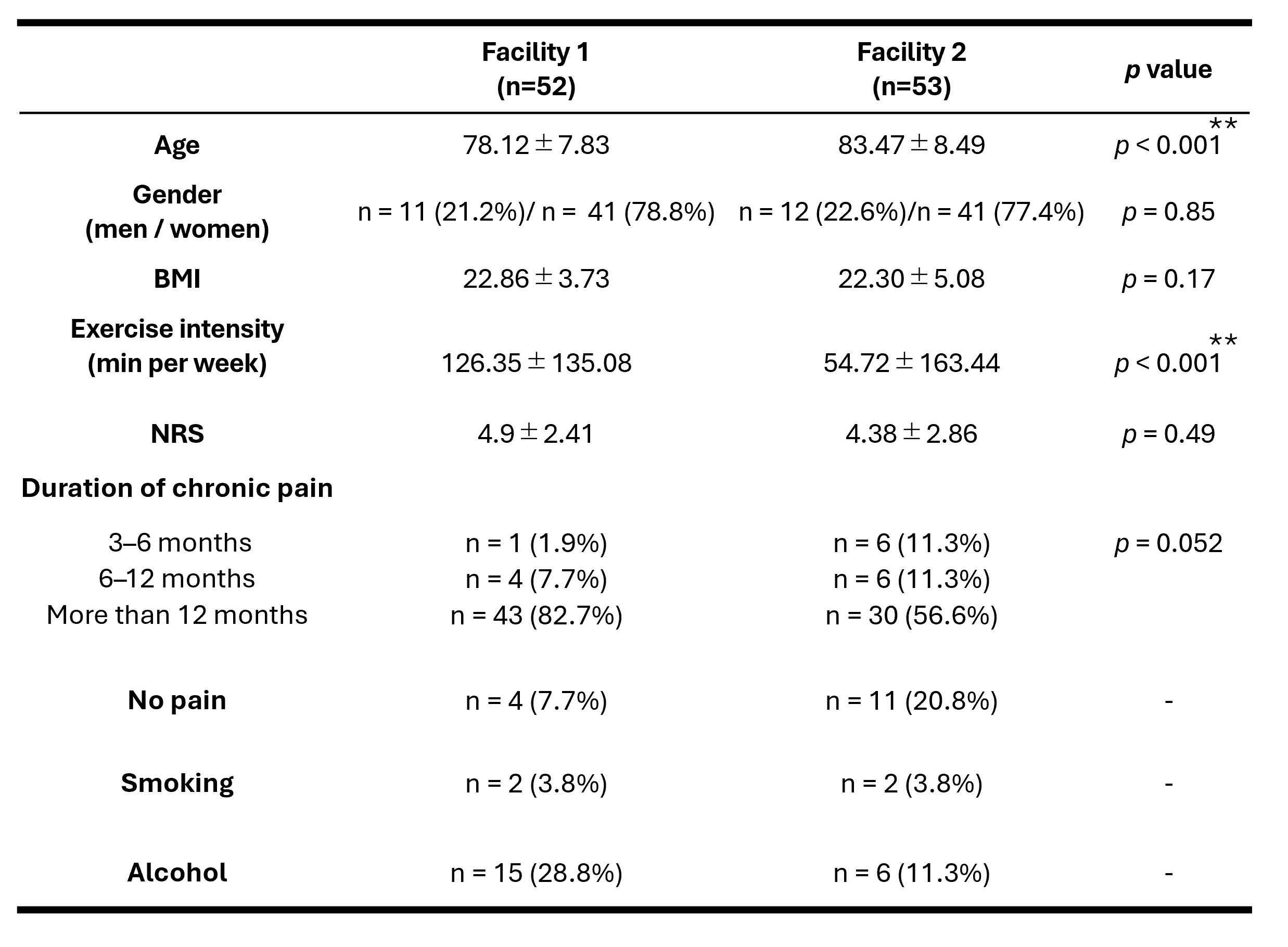

Supplement: S2 Table — The data for age, sex, BMI, exercise time per week, NRS, duration of pain, smoking, and alcohol in the two facilities are shown. Values are the means ± SD. The unpaired t-test was done to compare the two faculties. **p < 0.01. The distribution of male and female in the two facilities was analyzed using a chi-squared test. The distribution of pain duration in the two facilities was analyzed using a chi-squared test. (TIF) [file pone.0319647.s002.tif]

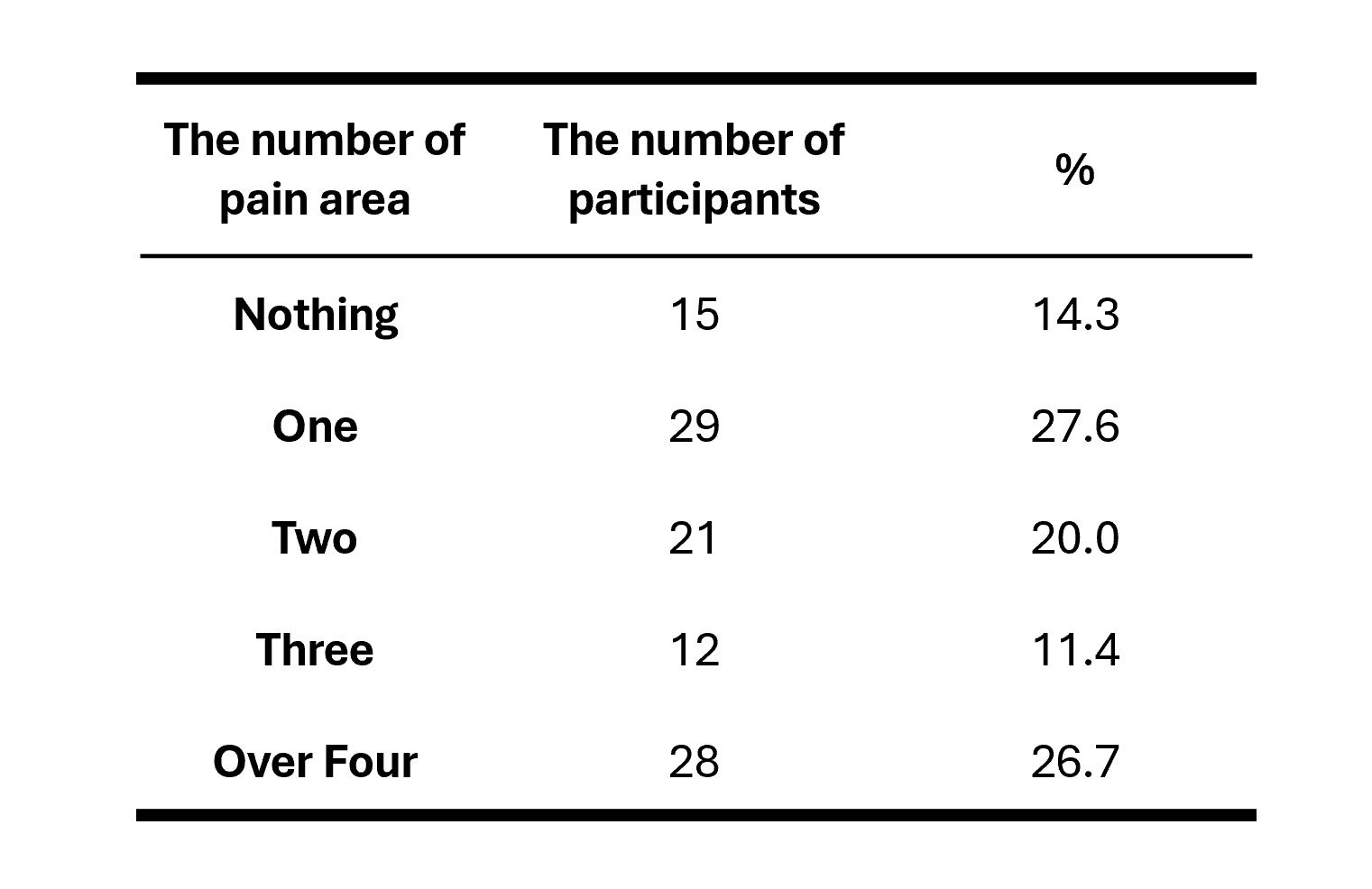

Supplement: S3 Table — The number of pain areas for each participant is counted. The number of participants with each number of pain areas is shown (n = 105). (TIF) [file pone.0319647.s003.tif]

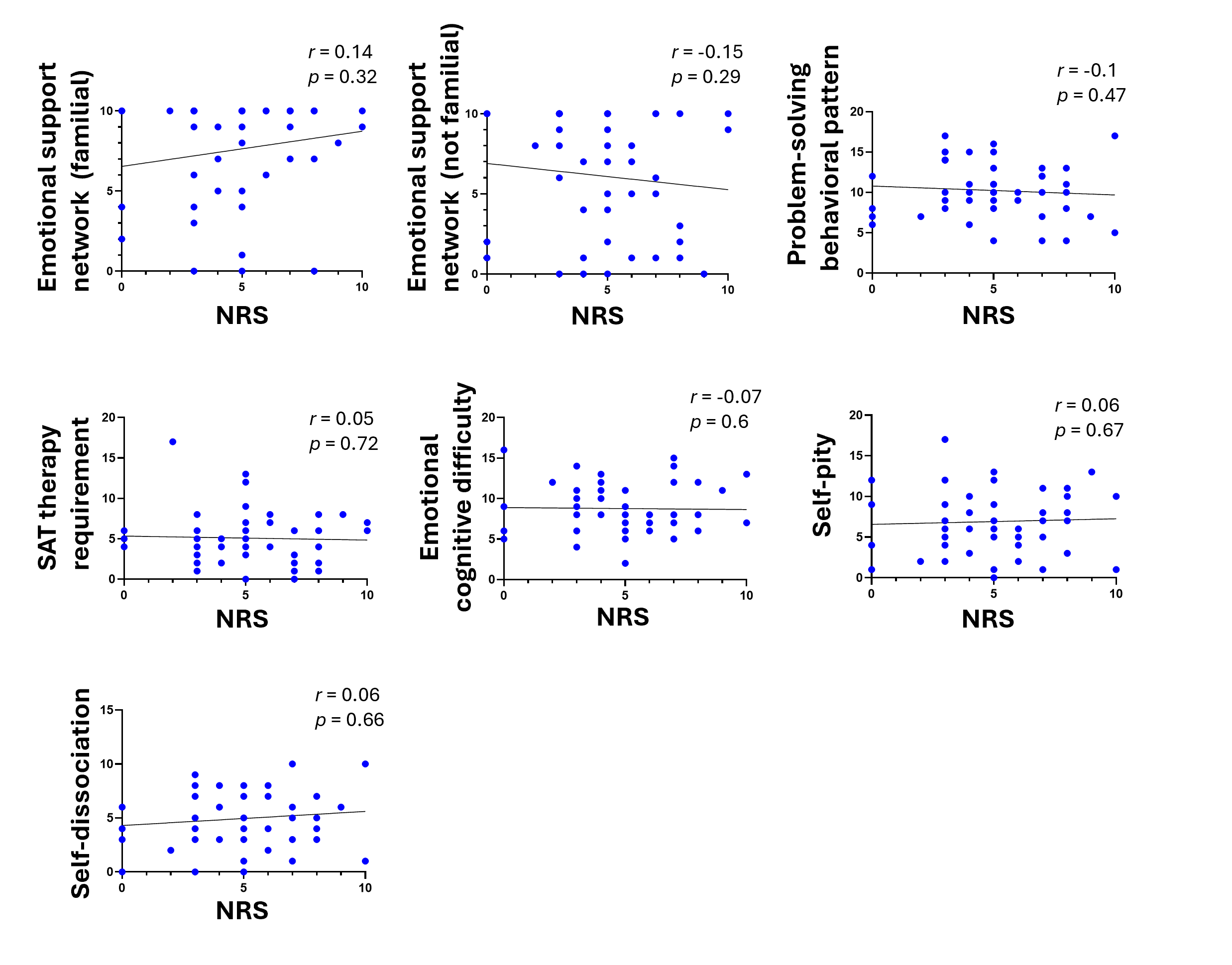

Supplement: S1 Fig — The correlations between the NRS score and each scale in SAT therapy (the emotional support network scale (familial or not familial), the problem-solving behavioral pattern scale, the SAT therapy requirement scale, the emotional cognitive difficulty scale, the self-pity scale, and the self-dissociation scale) are shown (n = 52). Spearman’s correlation coefficient was used. (TIF) [file pone.0319647.s004.tif]

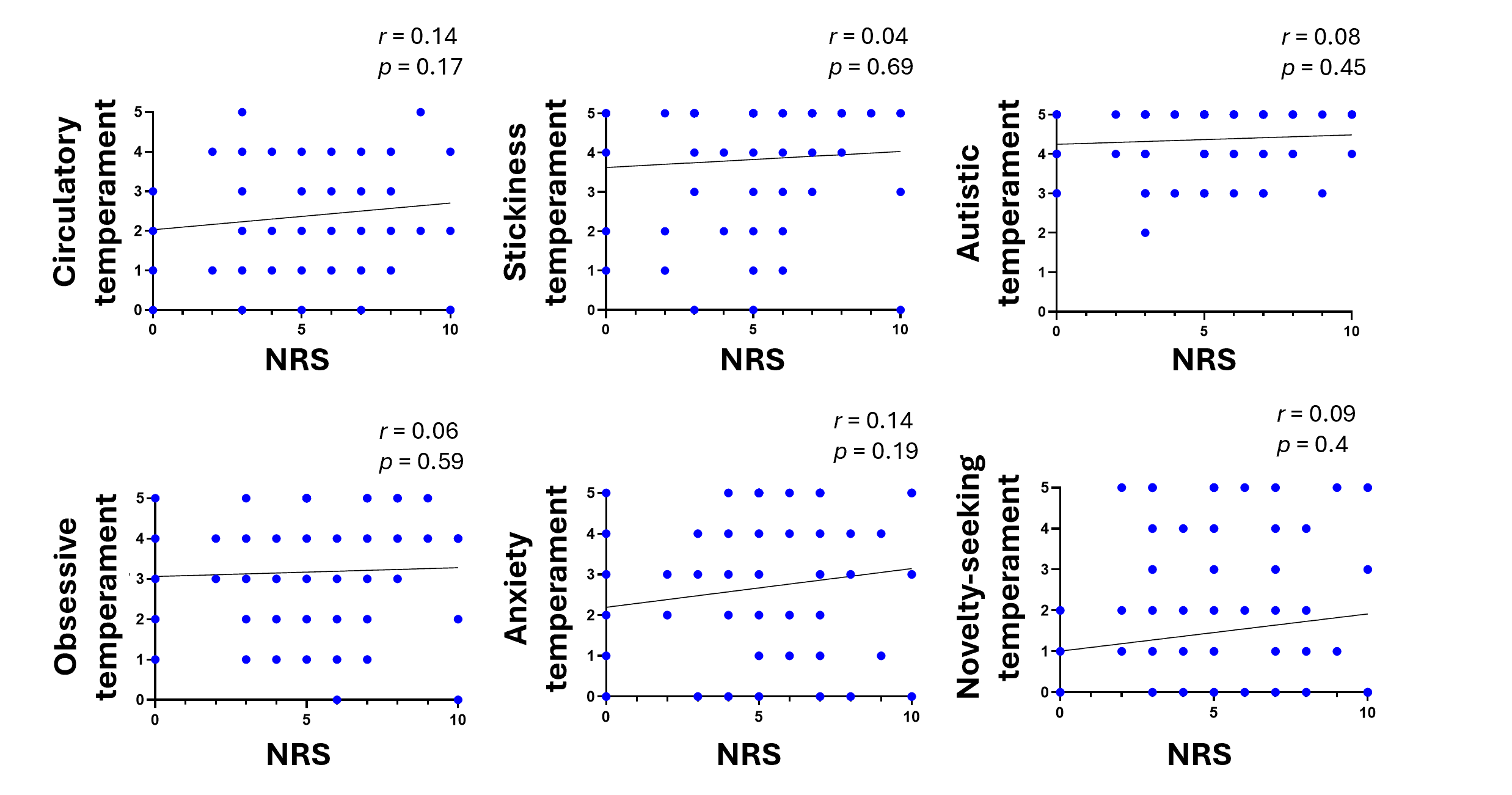

Supplement: S2 Fig — Correlations between the NRS score and each genetic character’s scale in SAT therapy (the circulatory temperament, the stickiness temperament, the autistic temperament, the obsessive temperament, the anxiety temperament, the novelty-seeking temperament) are shown (n = 92). Spearman’s correlation coefficient was used. (TIF) [file pone.0319647.s005.tif]

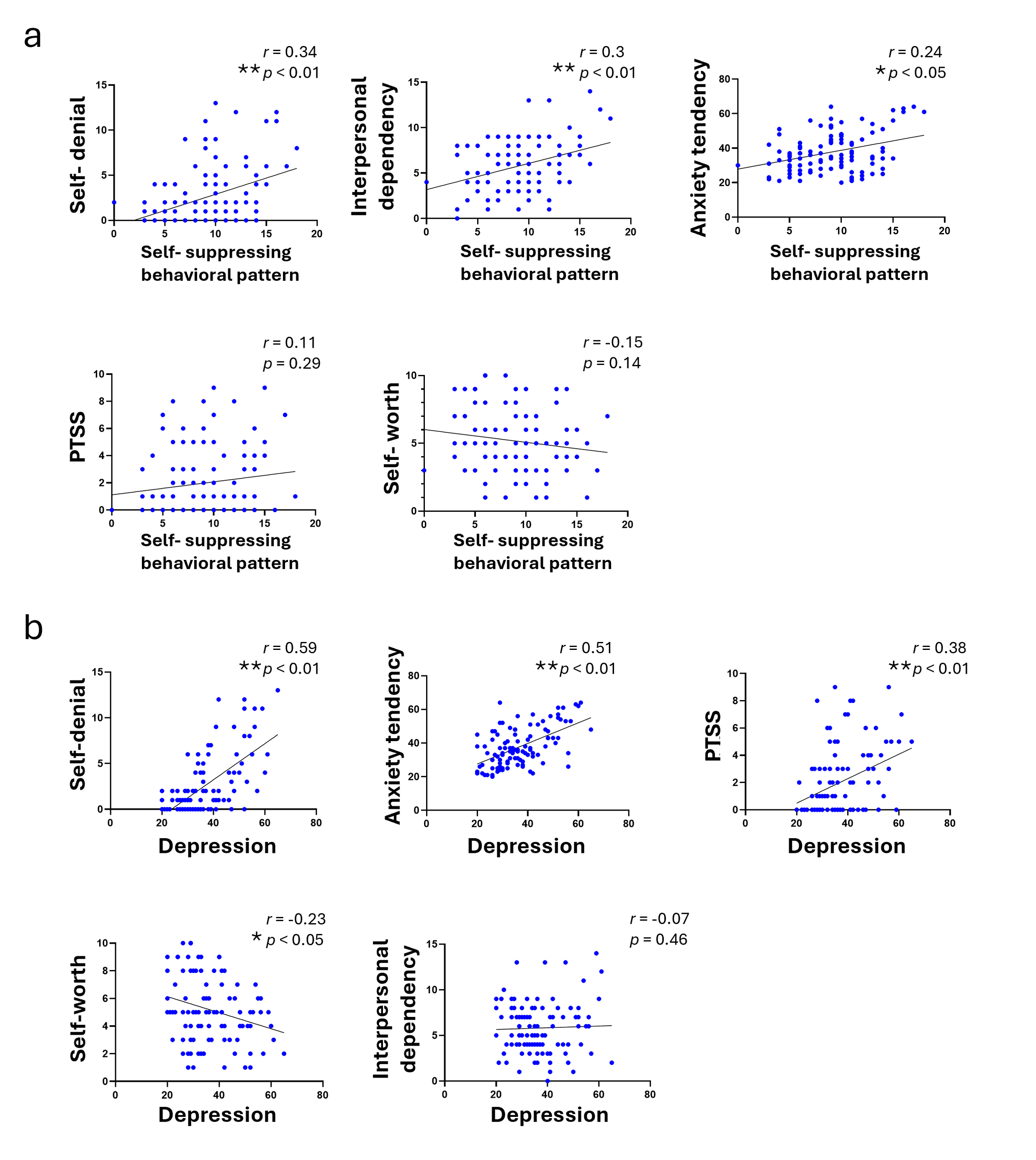

Supplement: S3 Fig — (a) Correlations between the self-suppressing behavioral pattern scale and each of the other SAT scales (except for the depression scale) (n = 105). Spearman’s correlation coefficient was used. * p < 0.05, **p < 0.01. (b) Correlations between the depression scale and each of the other SAT scales (except for the self-suppressing behavioral pattern scale) (n = 105). Spearman’s correlation coefficient was used. * p < 0.05, **p < 0.01. (TIF) [file pone.0319647.s006.tif]

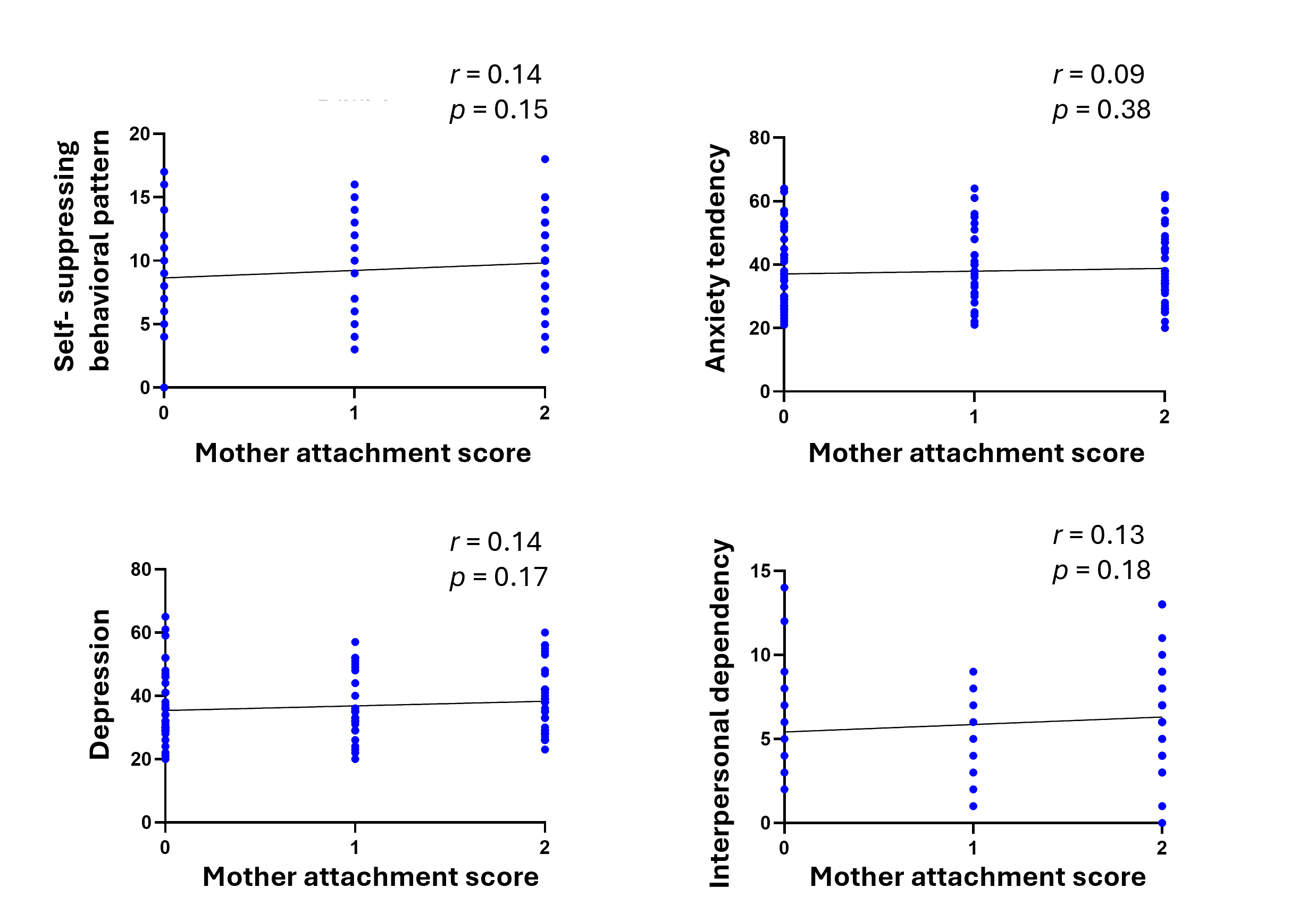

Supplement: S4 Fig — Correlations between the maternal attachment score and the SAT scales (the self-suppressing behavioral pattern scale, the anxiety tendency scale, the depression scale, and the interpersonal dependency scale) are shown (n = 101). Spearman’s correlation coefficient was used. Each score on the maternal attachment scale is as follows: 0 = enough attachment, 1 = a little attachment, 2 = poor attachment. (TIF) [file pone.0319647.s007.tif]
