# Supplementary material for: Self-suppressing behavioral patterns and depressive traits exacerbate chronic pain: Psychological trait assessment using the structured association technique method
Source: PLoS One. 2025 Mar 17;20(3):e0319647. doi: 10.1371/journal.pone.0319647 (PMC11913282; doi:10.1371/journal.pone.0319647)
Supplement: Attachment — Submitted filename: Responce to Reviewers.docx [file pone.0319647.s009.docx]

Reviewer 1

*Q1: Page 2, Line 8: Please provide specific values for the significant positive correlations.*

A1: We added the specific values in Page 2, Line 8-10.

*Q2: Page 6, Line 9: Please specify the inclusion and exclusion criteria.*

A2: We specified the inclusion and exclusion criteria in Page 6, Line 13-16.

*Q3:* *Page 8, Line 3: You mentioned "the preliminary analysis suggested no association with." Please support this with references.*

A3: The preliminary analysis was shown in S1 Fig. We added this reference in Page 8, Line 3-6.

*Q4:* *Page 10: There is insufficient demographic information about the participants. Is there more detailed information available?*

A4: We added demographic information in Table1, dividing the participants into without chronic pain group and chronic pain group (Page 10, Line 10 and Page 11, and Line 1-2). We also added the data of duration pain, smoking and alcohol in Table1 and S2 Table (Page 25, Line 30 and Page 26, and Line 1-4).

*Q5:* *Page 12, Line 10: "Fig 1" seems to be in the wrong position.*

A5: We wrote "Fig 1" in the correct position in Page 13, Line 9-13.

*Q6:* *Page 13, Line 12: Is there any missing content here?*

A6: We wrote the content of Fig 3 in Page 14, Line 18-19 and Page 15, Line 1.

*Q7: The study lacks a conclusion and a conflict of interest section.*

A7: We wrote a conclusion in Page 23, Line 10-16, and a conflict of interest section in Page 28, Line 10-12.

Reviewer 2

*Q1: Would you tell me about the specific physical diagnosis of the participants?*

A1: We added the physical diagnosis in Table 2 (Page 12, Line 1-7). The distribution of the diagnosed and not diagnosed groups in the high and low NRS groups were also analyzed using a chi-squared test.

*Q2: Would you tell me the definition of attachment in this study?*

A2: We added a description of attachment in Page 8, Line 9-11.
